# Furin Drives Colorectal Cancer Progression and Chemoresistance Through the TGF-β/ERK Signaling Pathway

**DOI:** 10.3390/cells15010043

**Published:** 2025-12-25

**Authors:** Pratheesh Kumar Poyil, Abdul K. Siraj, Sandeep Kumar Parvathareddy, Rafia Begum, Padmanaban Annaiyappa Naidu, Saravanan Thangavel, Khadija Alobaisi, Saud Azam, Fouad Al-Dayel, Khawla S. Al-Kuraya

**Affiliations:** 1 Experimental Therapeutics, Innovation & Research, King Faisal Specialist Hospital and Research Center, Riyadh 11211, Saudi Arabia; ppoyil@kfshrc.edu.sa (P.K.P.); brafia@kfshrc.edu.sa (R.B.); tsaravanan97@kfshrc.edu.sa (S.T.); 2 Translational Oncology Research, Innovation & Research, King Faisal Specialist Hospital and Research Center, Riyadh 11211, Saudi Arabia; asiraj@kfshrc.edu.sa (A.K.S.); kalobaisi@kfshrc.edu.sa (K.A.); 3 Human Cancer Genomic Research, Innovation & Research, King Faisal Specialist Hospital and Research Center, Riyadh 11211, Saudi Arabia; psandeepkumar@kfshrc.edu.sa (S.K.P.); pannaiyappanaidu97@kfshrc.edu.sa (P.A.N.); sjeelani@kfshrc.edu.sa (S.A.); 4 Department of Pathology, King Faisal Specialist Hospital and Research Center, Riyadh 11211, Saudi Arabia; dayelf@kfshrc.edu.sa

**Keywords:** colorectal cancer, furin, TGF-β/ERK signaling, chemoresistance, tumor progression

## Abstract

**Highlights:**

**What are the main findings?**

- Furin enhances colorectal cancer growth and contributes to resistance against 5-fluorouracil.
- A new furin–TGF-β–ERK signaling pathway is identified as a key driver of tumor aggressiveness.

**What are the implications of the main findings?**

- Inhibiting furin or its signaling pathway could improve treatment response in CRC.
- Furin may serve as a promising biomarker for identifying aggressive and chemoresistant colorectal tumors.

**Abstract:**

Colorectal cancer (CRC) remains one of the most lethal malignancies worldwide, with 5-fluorouracil (5-Fu) as a mainstay of treatment. However, intrinsic and acquired resistance to 5-Fu significantly limits therapeutic success. Furin, a proprotein convertase, is known to activate multiple substrates critical for tumor progression, yet its precise role in CRC remains unclear. In this study, we examined furin expression in a large cohort of CRC patient samples and performed functional analyses in CRC cell lines and xenograft models. Furin overexpression was seen in 46.9% (530/1131) of CRC cases and was significantly correlated with TGF-β and ERK1/2 activation. In vitro, induced furin overexpression enhanced proliferation and clonogenicity, accompanied by upregulation of TGF-β and ERK1/2 phosphorylation, whereas furin silencing attenuated tumor cell growth and TGF-β/ERK signaling. Manipulation of TGF-β revealed a reciprocal regulatory loop, whereby TGF-β upregulated furin expression, establishing a feed-forward circuit that augmented ERK signaling and tumor growth. Notably, 5-Fu-resistant CRC cell lines displayed elevated furin, TGF-β, and phospho-ERK1/2, while furin knockdown restored drug sensitivity. In vivo, furin overexpression enhanced tumor growth in xenografts, whereas its depletion markedly reduced tumor burden and TGF-β/ERK signaling activity. Collectively, these findings demonstrate that furin promotes CRC progression and chemoresistance through a positive feedback loop with TGF-β that sustains ERK activation. Targeting furin, alone or in combination with TGF-β/ERK inhibitors, may offer a promising therapeutic strategy for CRC.

## 1. Introduction

Colorectal cancer (CRC) is among the most prevalent malignancies worldwide and remains the second leading cause of cancer-related mortality in the United States [1,2]. In Saudi Arabia, CRC shows an increasing incidence and is currently recognized as the most common cancer among men [3]. Despite significant advances in therapeutic strategies, 5-Fluorouracil (5-Fu)-based combination chemotherapy continues to serve as the cornerstone of CRC treatment. However, the clinical benefit of 5-Fu is limited, with overall response rates of less than 15% due to the frequent development of intrinsic or acquired chemoresistance [4]. Such resistance not only diminishes treatment efficacy but also contributes to the development of liver metastases in nearly 50% of CRC patients, thereby complicating disease management and worsening clinical outcomes [5,6].

Furin, a calcium-dependent serine protease and a member of the pro-protein convertase family, is ubiquitously expressed across mammalian cells [7]. Together with other pro-protein convertases, furin is responsible for the proteolytic activation of diverse precursor proteins, many of which regulate biological processes critical for cancer initiation and progression [8,9]. Beyond its role in oncogenic pathways, furin also supports essential physiological functions, including normal development, immune regulation, and tissue homeostasis [9,10]. Through the maturation of substrates involved in proliferation, invasion, angiogenesis, and metastasis, furin has emerged as a pivotal player in tumorigenesis [11,12,13,14]. Indeed, aberrant furin expression has been documented across multiple malignancies, including breast cancer [15], lung cancer [16], head and neck [17], ovarian cancer [18], hepatocellular carcinoma [19], squamous cell carcinoma [20], sarcoma [8], and papillary thyroid carcinoma [21], as well as CRC [22]. Importantly, pharmacological or genetic inhibition of furin suppresses tumorigenic potential and metastatic progression in several cancer models [13,23,24,25,26], highlighting its therapeutic relevance.

Recent studies, including our own [21], have provided further evidence linking furin overexpression to poor prognosis [27]. We previously demonstrated that furin upregulation promotes papillary thyroid carcinoma progression and metastasis via the RAF/MEK signaling cascade [21]. Furin is well established as a key protease responsible for converting latent pro-TGF-β into its mature, bioactive form, thereby enabling downstream TGF-β signaling activation [28]. In parallel, numerous studies have shown that TGF-β can activate both canonical SMAD pathways and non-canonical MAPK/ERK cascades, processes that contribute to epithelial–mesenchymal transition, invasion, and metastatic progression in multiple cancers [29,30,31]. Despite these insights, the specific mechanistic contribution of furin to CRC progression and its potential role in modulating 5-Fu chemoresistance, particularly through the TGF-β/ERK axis, has not been fully elucidated. To the best of our knowledge, a reciprocal feed-forward regulatory loop between furin and TGF-β has not been documented in CRC or other solid tumors. Recent studies [22,32] have associated furin with *KRAS/BRAF*-driven colorectal tumorigenesis; however, these investigations did not demonstrate a reciprocal regulatory interaction between furin and TGF-β, nor did they connect furin activity to pathways governing 5-Fu resistance. Although TGF-β and MAPK signaling pathways have been implicated as important mediators of 5-Fu responsiveness in CRC [33,34,35], it remains unknown whether furin functions upstream of these pathways to orchestrate chemoresistance-associated signaling networks.

In the present study, we investigated the functional role of furin in CRC progression and its impact on resistance to 5-Fu chemotherapy. Using patient samples, in vitro assays, chemoresistant CRC models, and in vivo xenografts, we demonstrate that furin overexpression not only promotes CRC growth but also drives chemoresistance through a positive feedback loop with TGF-β, leading to sustained activation of ERK signaling. These findings identify furin as a critical driver of CRC aggressiveness and chemoresistance and suggest its potential as a therapeutic target to improve patient outcomes.

## 2. Materials and Methods

### 2.1. Sample Selection

Archival samples from 1131 CRC patients diagnosed between 1990 and 2018 at King Faisal Specialist Hospital and Research Center (Riyadh, Saudi Arabia) were included in the study. Formalin-fixed paraffin-embedded (FFPE) primary CRC samples (*n* = 1131) were retrospectively retrieved from the pathology archives of King Faisal Specialist Hospital and Research Centre between 1990 and 2018. Inclusion criteria required histologically confirmed CRC, availability of follow-up data, and sufficient tumor material for TMA construction.

Detailed clinicopathological data were noted from case records and have been summarized in Table 1. All samples were obtained from patients with approval from Institutional Review Board of the hospital. For the study, waiver of consent was obtained for archived paraffin tissue blocks from Research Advisory Council (RAC) under projects RAC#2190016 and RAC#2250020.

### 2.2. Tissue Microarray Construction and Immunohistochemistry

Tissue microarrays (TMAs) were constructed using tissue cores from all 1131 CRC samples. In addition, TMA of corresponding normal mucosa from 213 cases was also constructed. Normal mucosa were derived from tumor margins. For TMA construction, tissue cylinders with a diameter of 0.6 mm were punched from representative tumor regions of each donor tissue block and brought into recipient paraffin block using a modified semiautomatic robotic precision instrument (Beecher Instruments, Woodland, WI, USA). Two cores of CRC were arrayed from each case.

Standard protocol was followed for immunohistochemistry (IHC) staining [21]. For antigen retrieval, Dako (Dako Denmark A/S, Glostrup, Denmark) Target Retrieval Solution pH 9.0 (Catalog number S2367) was used, and the slides were placed in Pascal pressure cooker for 8 min at 120 °C. The slides were incubated with primary antibodies against furin (ab183495, Abcam, Cambridge, UK) with a dilution of 1:100 (pH 9.0), TGF-β (3C11, 1:2000, pH 6.0; Santa Cruz Biotechnology, Dallas, TX, USA) and phosphorylated ERK1/2 (pERK 1/2, 137F5, 1:1000, pH 6.0; Cell Signaling Technology, Danvers, MA, USA). The secondary detection was performed using the Dako EnVision+ System kit (Agilent Technologies, Glostrup, Denmark), with 3,3′-diaminobenzidine (Agilent Technologies, Glostrup, Denmark) serving as the chromogen. Slides were counterstained with hematoxylin, followed by dehydration, clearing, and mounting. Negative controls were prepared by omitting the primary antibody. To ensure consistency and reduce variability from slide aging, all staining was conducted simultaneously on freshly cut sections.

Furin, TGF-β and pERK 1/2 staining was scored using H score, as described previously [21,36]. Briefly, each TMA spot was assigned an intensity score from 0 to 3 (I0, I1–I3) and the proportion of tumor staining for that intensity was recorded as 5% increments from a range of 0–100 (P0, P1–P3). A final H score (range 0–300) was obtained by adding the sum of scores obtained for each intensity and proportion of area stained (H score = I1 × P1 + I2 × P2 + I3 × P3). X-tile software (version 3.6.1) [37] was used to define the optimal cut-off score. Based on X-tile plots, the cutoff H score for furin, TGF-β and pERK 1/2 were defined as 100, 80 and 60, respectively. Mismatch repair protein staining and evaluation was performed for MLH1, MSH2, MSH6 and PMS2 [38]. Loss of staining in cancer cells with concurrent positive staining in the nuclei of normal colon epithelial cells indicated protein inactivation. Cases showing loss of staining for at least one of the four proteins were labeled as microsatellite instability–high (MSI-H), whereas cases showing positive staining for all four proteins were labeled as microsatellite-stable (MSS).

### 2.3. Assessment of FURIN Genomic Alterations in the Cancer Genome Atlas (TCGA) Cohorts

Publicly available CRC datasets from TCGA were analyzed using the cBioPortal platform (https://www.cbioportal.org/, accessed on 9 September 2024) to assess the frequency and distribution of *FURIN* gene alterations. A total of 25 TCGA-derived CRC studies comprising 8201 patients were included based on the availability of complete somatic mutation and copy number alteration (CNA) data, standardized clinical annotations, and verified TCGA data quality. Both mutations and CNAs (amplifications and deletions) were extracted, and the overall proportion of *FURIN*-altered cases was calculated relative to the total CRC cohort size, providing a comprehensive overview of *FURIN* genomic events in CRC.

### 2.4. Reagents and Antibodies

Antibodies against furin (ab183495), TGF-β1 (155264), and Smad3 (ab28379) were obtained from Abcam Inc. (Cambridge, MA, USA). Phospho-Smad2 (3104), phospho-Smad3 (9520), pERK1/2 (4376), ERK1/2 (4695), and GAPDH (2118) antibodies were purchased from Cell Signaling Technology (Danvers, MA, USA). The Smad2 antibody (NBP2-37580) was obtained from Novus Biologicals (Centennial, CO, USA).

### 2.5. Tissue Culture Experiments

Human CRC cell lines HCT116, DLD1, and LOVO were obtained from the American Type Culture Collection (ATCC, Manassas, VA, USA) and maintained in RPMI-1640 medium supplemented with 10% fetal bovine serum (FBS), 100 U/mL penicillin, and 100 U/mL streptomycin at 37 °C in a humidified incubator with 5% CO_2_. Cell line authenticity was verified in-house using short tandem repeat profiling, and the results were consistent with published reference data [39,40]. Apoptosis analysis was performed using annexin V/propidium iodide (PI) dual staining and measured by flow cytometry as described previously [41].

### 2.6. Cell Lysis and Immunoblotting

Following treatment, CRC cells were lysed in phosphorylation lysis buffer containing 50 mM HEPES (pH 7.3), 150 mM NaCl, 1.5 mM MgCl_2_, 1.0 mM EDTA (pH 8.0), 100 mM NaF, 10 mM Na_2_H_2_P_2_O_7_, 200 μM Na_3_VO_4_, and 1× protease inhibitor cocktail (Roche Pharmaceuticals, Basel, Switzerland). Lysates were centrifuged at 14,000 rpm for 15 min at 4 °C, and protein concentrations were determined using the Bradford assay (Life Technologies, Carlsbad, CA, USA), a colorimetric method based on the binding of Coomassie Brilliant Blue dye to proteins [42]. Equal amounts of protein (30 μg) were resolved by SDS-PAGE, transferred to membranes, and immunoblotted with the indicated antibodies. Signals were detected using an enhanced chemiluminescence system (Amersham Biosciences, Arlington Heights, IL, USA) [43]. All uncropped Western blot images are presented in Supplementary Materials Figure S1.

### 2.7. Plasmid and Transfection

Plasmid constructs encoding human *FURIN* and *TGF-β1*, as well as shRNAs (short hairpin RNA) targeting human *FURIN* and *TGF-β1*, were obtained from Origene (Rockville, MD, USA). Transient transfections were performed using Lipofectamine™ 2000 (Invitrogen, Carlsbad, CA, USA) following the manufacturer’s protocol. Briefly, cells were seeded in 6-well plates and transfected with 4 μg of plasmid DNA at ~50% confluence. 48 h post-transfection, stable overexpression clones were selected with G418, whereas stable knockdown clones were established using puromycin. Successful overexpression and silencing of furin and TGF-β1 were confirmed by immunoblotting [44].

### 2.8. Generation of 5-Fu-Resistant (5-FuR) CRC Cell Lines

To investigate the role of furin in chemoresistance, HCT116, DLD1, and LOVO cell lines were cultured in RPMI-1640 medium supplemented with 10% FBS and 1% penicillin-streptomycin under standard conditions (37 °C, 5% CO_2_). Chemoresistant sublines were generated by exposing parental cells to increasing concentrations of 5-Fu (1 µM → 2 µM → 5 µM → 10 µM → 25 µM → 50 µM) over several weeks until they exhibited stable resistance. Resistance was confirmed by 3-(4,5-dimethylthiazol-2-yl)-2,5-dimethyltetrazolium bromide (MTT) assay and apoptosis assay.

### 2.9. MTT Cell Viability and IC_50_ Determination

Cell viability was assessed using the MTT assay [45]. Parental and 5-FuR CRC cells (HCT116, DLD1, and LOVO) were seeded in 96-well plates at a density of 5 × 10^3^ cells per well and allowed to attach overnight. Cells were then treated with a range of 5-Fu concentrations (0–200 μM) for 48 h. MTT reagent (5 mg/mL) was added to each well and incubated for 4 h at 37 °C. Formazan crystals were dissolved in DMSO, and absorbance was measured at 570 nm using a microplate reader. Dose–response curves were generated, and IC_50_ values were determined by interpolating the drug concentration corresponding to 50% cell viability. All experiments were performed with six replicates and independently repeated at least three times. Detailed IC_50_ values for parental and 5-FuR cell lines are summarized in Supplementary Table S1 (*n* = 3).

### 2.10. Clonogenic Assay

Following the respective treatments (5-Fu exposure at indicated doses and durations, stable furin knockdown, furin overexpression or corresponding controls), cells were trypsinized and counted using trypan blue exclusion, and 500 viable cells/well were seeded in triplicate in 6-well plates with drug-free complete medium. After overnight attachment at 37 °C in 5% CO_2_, cells were cultured undisturbed for 8–10 days, with one medium replacement on day 4–6. At the endpoint, colonies were fixed in 4% formaldehyde and subsequently stained with 2% crystal violet dissolved in 10% methanol. Colony numbers were manually counted under an inverted microscope by two independent blinded investigators, and representative images were captured for documentation.

### 2.11. Animals and Xenografts Study

Six-week-old female nude mice (NU/J) were obtained from Jackson Laboratories (Bar Harbor, ME, USA) and acclimatized for at least one week in a sterile, pathogen-free facility under controlled conditions (12 h light/12 h dark cycle) with free access to food and water. All animal experiments were conducted in accordance with institutional regulations and were approved by the Animal Care and Use Committee (ACUC) of King Faisal Specialist Hospital and Research Center (RAC#2250020; approval date: 18 August 2025). For xenograft generation, DLD1 cells (4 × 10^6^ per mouse) were suspended in serum-free medium mixed 1:1 with Matrigel basement membrane matrix and subcutaneously implanted into the flanks of NU/J mice (*n* = 5). Tumor volume and body weight were monitored weekly to assess tumor growth and overall health. At the completion of week 4 post-inoculation, mice were euthanized by CO_2_ inhalation followed by cervical dislocation; tumors were excised and weighed, and tissues were snap-frozen in liquid nitrogen for downstream analyses.

### 2.12. Statistical Analysis

The associations between clinico-pathological variables and protein expression was performed using contingency table analysis and Chi square tests. Two-sided tests were used for statistical analyses with a limit of significance defined as *p* value < 0.05. Data analyses was performed using the JMP14.0 (SAS Institute, Inc., Cary, NC, USA) software package. *p*-values were calculated using the Chi-square test by comparing clinico-pathological variables between furin-overexpressing and furin-low expressing CRC cases.

For all functional studies, Data represent three independent biological experiments (*n* = 3), with technical replicates specified for each assay. For multiple group comparisons, one-way analysis of variance (ANOVA) was performed using IBM SPSS Statistics version 21 (IBM Corp., Armonk, NY, USA). A *p*-value < 0.05 was considered statistically significant.

## 3. Results

### 3.1. Furin Expression in CRC and Its Clinico-Pathological Associations

**Figure 1:**
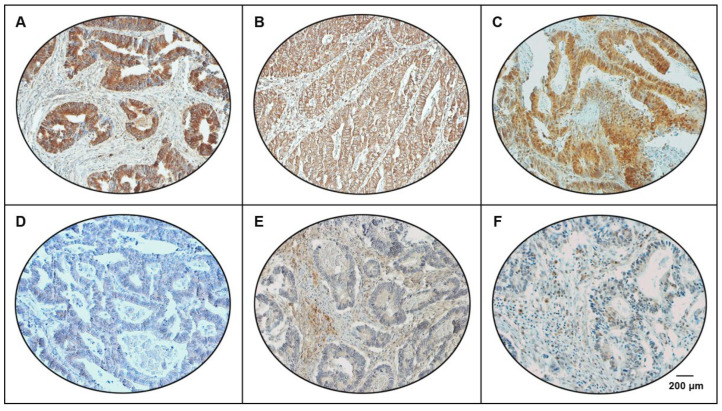
Tissue microarray (TMA) based immunohistochemistry analysis of furin, TGF-β1 and pERK1/2 in CRC patients. CRC TMA spots showing overexpression of (**A**) furin, (**B**) TGF-β1 and (**C**) pERK1/2. In contrast, another set of TMA spots showing reduced expression of (**D**) furin, (**E**) TGF-β1 and (**F**) pERK1/2. 20 X/0.70 objective on an Olympus BX 51 microscope (Olympus America Inc., Center Valley, PA, USA, scale bar = 200 μm).

Furin overexpression was noted in 46.9% (530/1131) of CRC cases (Figure 1). Matched normal colonic mucosa were available for 213 cases and used as an internal control to assess baseline Furin expression (Supplementary Figure S2). A significant association was noted between furin overexpression and older age at diagnosis of CRC (>50 years; *p* = 0.0156) (Table 1), but no significant association was noted with other clinico-pathological characteristics. However, furin overexpression was found to be associated with pERK1/2 (*p* < 0.0001) and TGFβ1 (*p* < 0.0001) (Figure 1, Table 1). Furin overexpression was significantly enriched in *KRAS*-mutant tumors (*p* < 0.0001), but no significant association was found with *BRAF*-mutant status (*p* = 0.0873) (Table 1).

### 3.2. FURIN Genomic Landscape in CRC

Analysis of 8201 CRC patient samples from 25 TCGA studies revealed that *FURIN* is altered in a subset of CRC cases. Overall, *FURIN* mutations were identified in 0.79% of cases (65/8201), while CNAs (amplifications and deletions) were detected in 0.20% of cases (16/8201). These alterations were distributed across the analyzed CRC cohorts, with no single dataset showing a high frequency of *FURIN* genomic events.

### 3.3. Furin Promotes CRC Cell Growth via TGF-β/ERK1/2 Signaling

**Figure 2:**
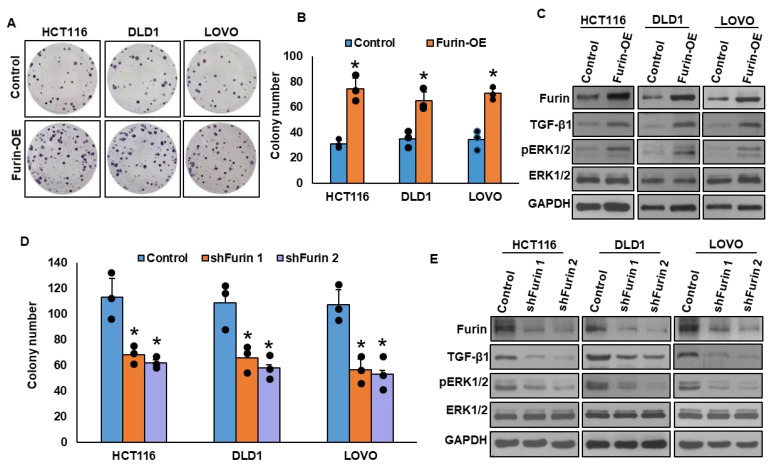
Furin promotes CRC cell growth through TGF-β/ERK1/2 signaling. (**A**,**B**) Furin overexpression (Furin-OE) enhances clonogenicity. CRC cells transfected with furin cDNA were selected, and stable clones were subjected to clonogenic assays (*n* = 3). (**C**) Furin overexpression activates TGF-β/ERK1/2 signaling. Protein lysates from furin-overexpressing clones were analyzed by immunoblotting. (**D**) Furin knockdown suppresses clonogenicity. CRC cells transfected with two independent furin shRNA constructs were subjected to clonogenic assays (*n* = 3). (**E**) Silencing furin decreases TGF-β expression and ERK1/2 phosphorylation. * *p* < 0.05 compared with controls.

In our CRC patient cohort, furin overexpression was significantly associated with elevated TGF-β1 expression and ERK1/2 phosphorylation (Table 1). Functional validation using three CRC cell lines (HCT116, DLD1, and LOVO) further confirmed these findings. As shown in Figure 2A,B, furin overexpression markedly enhanced clonogenic growth, indicating its strong proliferative potential. This growth advantage was accompanied by a marked increase in TGF-β1 expression and ERK1/2 phosphorylation (Figure 2C), consistent with activation of the non-canonical TGF-β1/ERK pathway. In contrast, stable furin knockdown significantly suppressed colony formation (Figure 2D) and led to a marked reduction in both TGF-β1 expression and ERK1/2 activation (Figure 2E), demonstrating the functional dependency of CRC cells on furin-driven signaling.

To further explore whether furin also regulates the canonical branch of TGF-β signaling, we examined SMAD2 and SMAD3 phosphorylation. As shown in Supplementary Figure S3, furin overexpression increased phosphorylation of both SMAD2 and SMAD3, thereby confirming activation of the canonical TGF-β/SMAD signaling cascade. Conversely, furin silencing attenuated SMAD2/3 phosphorylation, suggesting that furin simultaneously regulates both canonical (SMAD2/3-dependent) and non-canonical (ERK1/2-dependent) TGF-β signaling.

### 3.4. A Positive Feedback Loop Between Furin and TGF-β Promotes CRC Cell Growth

**Figure 3:**
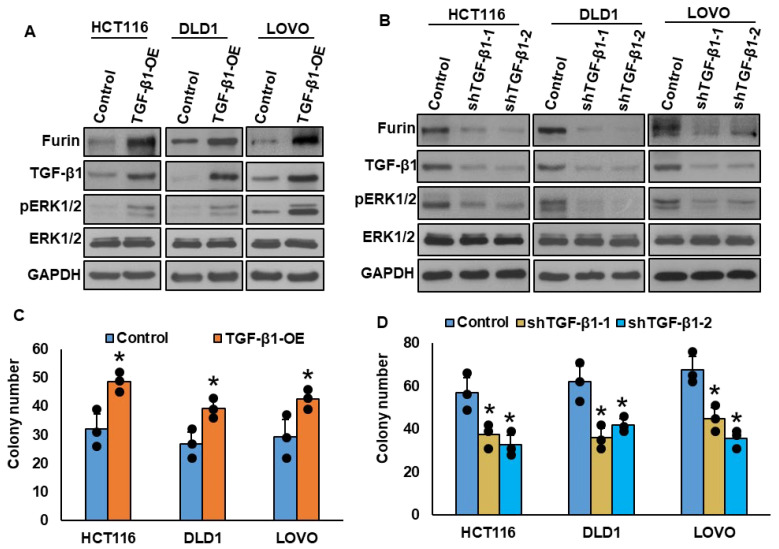
A positive feedback loop between furin and TGF-β promotes CRC cell growth. (**A**) TGF-β1 overexpression upregulates furin and enhances ERK1/2 phosphorylation. CRC cells transfected with TGF-β1 cDNA were analyzed by immunoblotting. (**B**) TGF-β1 knockdown decreases furin expression and ERK1/2 activation. Protein lysates from CRC cells transfected with two independent TGF-β1 shRNA constructs were examined by immunoblotting. (**C**) Overexpression of TGF-β1 increases clonogenic growth. Stable TGF-β1-overexpressing CRC clones were subjected to clonogenic assays (*n* = 3). (**D**) TGF-β1 silencing reduces clonogenic growth. CRC cells transfected with two distinct TGF-β1 shRNAs were analyzed by clonogenic assays (*n* = 3). * *p* < 0.05 compared with controls.

Furin is a well-established activator of TGF-β, mediating cleavage of its precursor (pro-TGF-β) within the trans-Golgi network [46]. To investigate whether a reciprocal regulatory relationship exists, we manipulated TGF-β1 expression in CRC cell lines. Overexpression of TGF-β1 markedly increased furin expression and enhanced ERK1/2 activation (Figure 3A), while TGF-β1 knockdown produced the opposite effect (Figure 3B). Functionally, TGF-β1 overexpression promoted cell proliferation, as shown by increased colony number (Figure 3C), whereas its silencing suppressed cell growth (Figure 3D). These results indicate the presence of a positive feedback loop between furin and TGF-β, activating ERK1/2 signaling and promoting CRC progression.

### 3.5. Furin Drives CRC Chemoresistance Through the TGF-β/ERK Signaling Pathway

**Figure 4:**
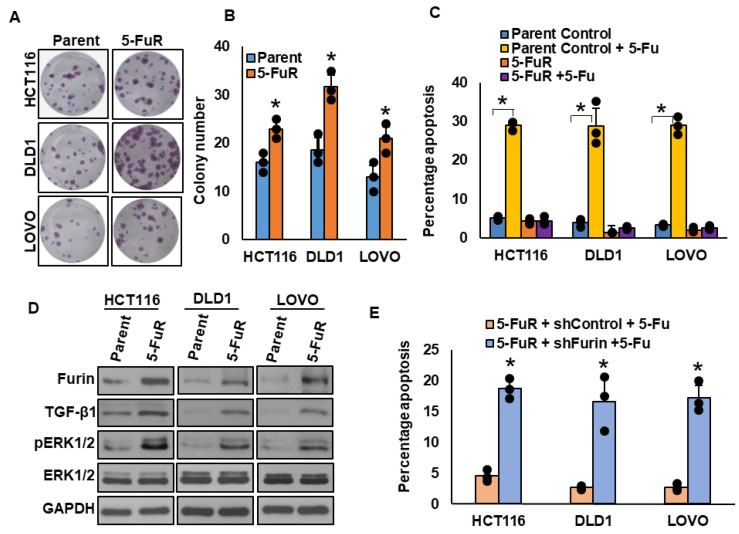
Furin drives CRC chemoresistance through the TGF-β/ERK signaling pathway. (**A**,**B**) The 5-FuR CRC cell lines exhibit enhanced proliferation. Parental and 5-FuR cells were subjected to clonogenic assays (*n* = 3). (**C**) The 5-FuR CRC cell lines show complete resistance to 5-Fu treatment (50 μM), as assessed by Annexin V/PI apoptosis analysis (*n* = 3). (**D**) The 5-FuR cells display increased expression of furin, TGF-β1, and ERK1/2 phosphorylation, as determined by immunoblotting. (**E**) Furin knockdown restores 5-Fu sensitivity (50 μM), as shown by apoptosis analysis (*n* = 3). * *p* < 0.05 compared with controls.

High furin expression correlated with aggressive clinicopathological features in CRC patients. To investigate its role in chemoresistance, we generated 5-Fu-resistant (5-FuR) derivatives of HCT116, DLD1, and LOVO cells by stepwise drug exposure. Compared with their parental counterparts, 5-FuR cell lines displayed enhanced proliferation (Figure 4A,B) and complete resistance to 5-Fu treatment (Figure 4C). Immunoblotting revealed elevated furin and TGF-β1 expression, along with increased ERK1/2 phosphorylation in resistant cells (Figure 4D). Notably, furin silencing restored sensitivity to 5-Fu (Figure 4E). These findings demonstrate that furin drives CRC chemoresistance via the TGF-β/ERK axis and highlight its potential as a therapeutic target.

### 3.6. Furin Promotes Tumor Growth In Vivo

**Figure 5:**
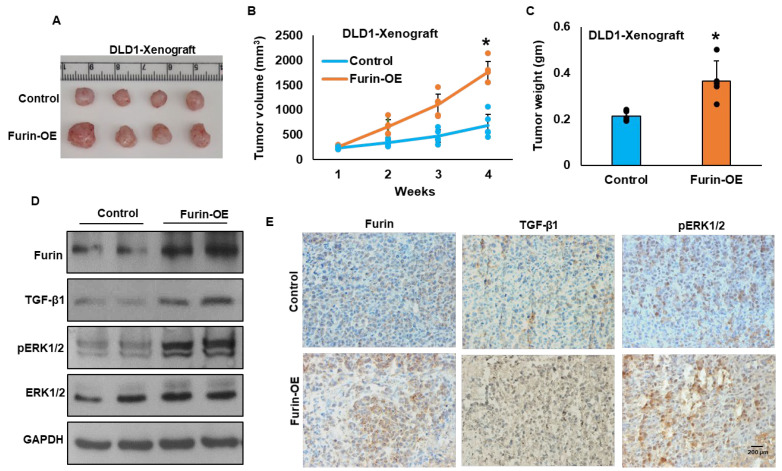
Furin overexpression enhances tumor growth in vivo. (**A**) Representative images showing the gross morphology and comparative size of excised xenograft tumors from mice. Although five animals were used per group, one tumor was inadvertently damaged during excision and could not be presented as an intact representative image. Importantly, tumor volume and weight measurements were obtained from all five animals, and quantitative analyses include the full cohort. Briefly, DLD1 cells stably expressing either an empty vector or *FURIN* cDNA were injected subcutaneously into the right flanks of NU/J mice (*n* = 5). (**B**) Tumor volume and body weight were recorded weekly to monitor growth and animal condition. (**C**) After four weeks, mice were euthanized, and the tumors were collected and weighed. Data are shown as mean ± SD (*n* = 5). (**D**) Tumor lysates were analyzed by immunoblotting with antibodies against Furin, TGF-β1, pERK1/2, total ERK1/2, and GAPDH. (**E**) Immunohistochemical staining of tumor sections (5 µm) was performed using antibodies against Furin (ab183495, Abcam, 1:100), TGF-β (3C11, Santa Cruz Biotechnology, 1:2000), and pERK1/2 (137F5, Cell Signaling Technology, 1:1000). Staining was developed using the Dako EnVision+ System with 3,3′-diaminobenzidine as the chromogenic substrate. Scale bar = 200 μm. Endpoint tumor volumes and weight were compared using one-way ANOVA; * *p* < 0.05 was considered statistically significant.

Having demonstrated a growth-promoting role for furin in CRC cells in vitro, we next sought to validate these findings in vivo. DLD1 cells stably overexpressing furin, along with empty vector-transfected controls, were subcutaneously implanted into the flanks of NU/J mice (*n* = 5). After four weeks, the animals were euthanized, and tumor tissues were collected for further analysis. Furin overexpression markedly enhanced tumor progression, as evidenced by significant increases in both tumor volume (Figure 5A,B) and tumor weight (Figure 5C). Consistently, immunoblotting of tumor lysates revealed elevated expression of furin and TGF-β1, together with enhanced ERK1/2 phosphorylation in furin-overexpressing tumors (Figure 5D). Immunohistochemical staining further confirmed a higher abundance of Furin, TGF-β1, and phospho-ERK1/2-positive cells in these tumors (Figure 5E).

**Figure 6:**
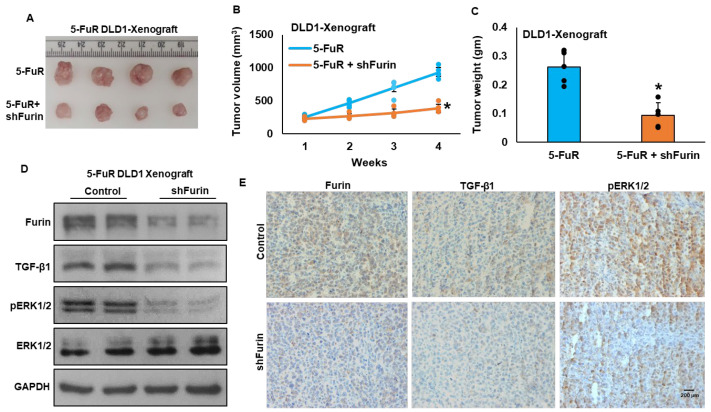
Depletion of furin inhibits tumor growth in vivo. (**A**) Representative images illustrating the size and appearance of tumors harvested from mice following the experimental endpoint. Although five animals were used per group, one tumor was inadvertently damaged during excision and could not be presented as an intact representative image. Importantly, tumor volume and weight measurements were obtained from all five animals, and quantitative analyses include the full cohort. Briefly, 5-FuR DLD1 cells expressing either an empty vector or *FURIN*-targeting shRNA were injected subcutaneously into the right flanks of NU/J mice (*n* = 5). (**B**) Tumor volume and body weight were assessed weekly to track tumor progression and overall health. (**C**) After four weeks, mice were euthanized, and tumors were collected and weighed. Data are shown as mean ± SD (*n* = 5). (**D**) Tumor lysates were analyzed by immunoblotting with antibodies against Furin, TGF-β1, pERK1/2, total ERK1/2, and GAPDH. (**E**) Immunohistochemical staining of tumor sections (5 µm) was performed using antibodies against Furin, TGF-β, and pERK1/2. Staining was developed using the Dako EnVision+ System with 3,3′-diaminobenzidine as the chromogenic substrate. Scale bar = 200 μm. Endpoint tumor volumes and weight were compared using one-way ANOVA; * *p* < 0.05 was considered statistically significant.

Conversely, depletion of furin in the 5-FuR DLD1 cell line led to pronounced delay of tumor growth in NU/J mice, as reflected by reduced tumor volume (Figure 6A,B) and weight (Figure 6C). Knockdown of furin also led to decreased expression of furin, TGF-β1, and pERK1/2 in tumor lysates (Figure 6D), accompanied by only a few positively stained cells in immunohistochemical analyses (Figure 6E). Taken together, these results provide compelling in vivo evidence that furin promotes CRC tumor growth, at least in part through the TGF-β/ERK signaling pathway, underscoring its potential as a therapeutic target to limit tumor progression and chemoresistance.

## 4. Discussion

In this study, we provide convincing evidence that furin acts as a critical driver of CRC progression and chemoresistance, functioning at least in part through activation of the TGF-β/ERK signaling pathway. Our findings demonstrate that furin overexpression enhances CRC cell proliferation, promotes chemoresistance to 5-Fu, and facilitates tumor growth in vivo. Importantly, we identify a positive feedback loop between furin and TGF-β, which sustains ERK activation and contributes to CRC aggressiveness. These results establish furin not only as a potential prognostic biomarker but also as a potential therapeutic target in CRC.

The observation that nearly half of CRC cases exhibit furin overexpression underscores its clinical relevance. Notably, genetic alterations in *FURIN* are relatively uncommon in CRC, indicating that furin upregulation is more likely driven by transcriptional or post-transcriptional mechanisms rather than frequent somatic mutations or copy number changes. Consistent with previous reports implicating furin in the activation of oncogenic substrates such as growth factors, adhesion molecules, and matrix metalloproteinases [11,13], our findings reveal a previously unrecognized mechanistic link between furin and oncogenic TGF-β signaling in CRC. Specifically, furin overexpression markedly increased TGF-β levels and enhanced ERK1/2 phosphorylation in both patient tissues and in vitro models, whereas furin silencing led to a concomitant reduction in TGF-β expression and pERK1/2 levels. These data demonstrate that furin potentiates non-canonical TGF-β/ERK signaling, thereby promoting CRC cell proliferation, survival, and chemoresistance. In parallel, furin also positively regulated canonical TGF-β signaling, as evidenced by increased SMAD2 and SMAD3 phosphorylation upon furin overexpression and their suppression following furin knockdown. Collectively, these findings position furin as a central driver of CRC progression through coordinated activation of both non-canonical (ERK1/2) and canonical (SMAD2/3) TGF-β signaling pathways. This regulatory axis highlights furin’s critical role in amplifying oncogenic TGF-β networks and reinforces its potential as a therapeutic target in colorectal cancer.

This study provides novel mechanistic insights into the oncogenic role of furin in CRC. Unlike previous reports that primarily associated furin activity with the processing of general oncogenic substrates or *KRAS/BRAF*-driven tumorigenesis [21,22,32], our study uniquely identifies a positive feedback loop between furin and TGF-β that sustains ERK activation and drives both tumor progression and 5-Fu resistance. This reciprocal regulation between furin and TGF-β has not been previously described in CRC or other solid tumors. Furthermore, by integrating clinical data from a large patient cohort, in vitro functional assays, and in vivo xenograft models, our work provides a comprehensive demonstration of furin’s dual role in CRC progression and therapeutic resistance. These findings offer a new perspective on furin as both a prognostic biomarker and a promising combinatorial target with TGF-β/ERK inhibitors in CRC management.

While previous studies have reported elements of a TGF-β–furin positive feedback loop in hepatocellular carcinoma [47] and glioblastoma [48], CRC-specific mechanistic evidence for a reciprocal loop has been lacking. Importantly, none of these studies demonstrated a reciprocal regulatory loop between TGF-β and furin specifically in CRC cells. Our findings extend this concept by providing, to our knowledge, the first demonstration of a bidirectional TGF-β–furin regulatory loop in CRC, which sustains ERK activation and enhances proliferative and chemoresistant phenotypes. Thus, our work refines and contextualizes earlier observations by defining this feedback mechanism specifically in colorectal cancer biology.

Chemoresistance remains a major obstacle in CRC management, with 5-Fu resistance significantly reducing therapeutic efficacy and contributing to poor patient outcomes. Our results demonstrate that furin expression is markedly elevated in 5-FuR CRC cell lines, accompanied by enhanced TGF-β signaling and ERK phosphorylation. Silencing furin not only suppressed tumor growth, but also restored sensitivity to 5-Fu, directly implicating furin as a driver of chemoresistance. These findings are consistent with prior reports showing that pharmacological inhibition of furin or proprotein convertases can attenuate malignant properties and may sensitize CRC cells to therapeutic agents [32,49], though direct evidence in 5-Fu-resistant CRC remains limited.

While furin has long been recognized as a key activator of latent TGF-β precursors [46], evidence for the reciprocal regulation of furin by TGF-β is limited. Our data provide the first demonstration, to our knowledge, of a positive feedback loop between furin and TGF-β in CRC, which sustains ERK signaling and promotes tumor progression. Such a feed-forward mechanism likely amplifies oncogenic signaling, driving tumor progression and resistance to therapy. This finding provides new insights into the complex regulation of the TGF-β axis in CRC and suggests that dual targeting of furin and TGF-β may represent an effective therapeutic strategy.

An important contextual factor influencing these findings is the mutational background of the CRC cell lines used in this study. All CRC cell lines used in this study harbor activating *KRAS* mutations, an oncogenic context previously shown to influence dependency on furin-mediated processing [22]. Consistent with our CRC patient data analysis, which demonstrated significantly higher furin expression in *KRAS*-mutant tumors, these findings suggest that *KRAS*-driven signaling may sensitize CRC cells to furin inhibition, thereby increasing the chemosensitizing and antiproliferative effects observed in our experiments. Although our results are highly consistent with prior work reporting preferential vulnerability of *KRAS*- or *BRAF*-mutant CRC cells to furin depletion [22], validation in *BRAF*-mutant and wild-type models will be essential to fully define the molecular dependency.

Our in vivo xenograft experiments further validate the role of furin in tumor progression, with furin overexpression promoting rapid tumor growth and furin depletion significantly suppressing tumor development. Importantly, the reduction in tumor burden following furin knockdown was associated with decreased TGF-β and pERK1/2 expression, confirming the central role of this signaling cascade in mediating furin-driven oncogenesis. Based on the well-established roles of TGF-β signaling in regulating stromal remodeling, immune modulation, and angiogenesis, it is plausible that furin-mediated activation of TGF-β may contribute to these processes; however, these effects were not directly assessed in the present study. In addition to promoting proliferation and chemoresistance, furin may also facilitate CRC invasion, metastasis, and tumor microenvironment remodeling. By activating substrates such as TGF-β [28], MMPs and integrins [50], furin can enhance extracellular matrix degradation, epithelial–mesenchymal transition, and stromal activation, thereby supporting tumor progression. These findings suggest that targeting the furin–TGF-β/ERK signaling axis could not only inhibit tumor growth but also reduce metastatic potential and improve therapeutic response in CRC.

From a translational perspective, our results suggest that furin represents an attractive therapeutic target for CRC. Several furin inhibitors, including peptide-based and small-molecule compounds, have been developed and shown efficacy in preclinical cancer models [13,23]. However, their clinical application remains limited by concerns about toxicity and specificity, given the broad physiological roles of furin. The identification of the furin–TGF-β/ERK axis as a tumor-promoting pathway provides opportunity for combinatorial treatments, targeting furin and TGF-β or ERK simultaneously, and improving therapeutic benefit whilst reducing off-target effects. Moreover, furin protein expression could serve as a biomarker to stratify patients likely to benefit from such interventions. In this context, future studies employing genetic or pharmacological inhibition of TGF-β signaling in furin-overexpressing CRC models will be valuable to further delineate the pathway-specific contribution of TGF-β/ERK signaling to furin-driven tumor growth and chemoresistance.

Nevertheless, our study has some limitations. Although we established a strong link between furin, TGF-β, and ERK signaling, the precise downstream substrates of furin that contribute to chemoresistance remain to be fully elucidated. Future studies employing proteomic profiling and in vivo models of metastasis may help to uncover additional pathways regulated by furin. Furthermore, while our findings suggest therapeutic potential, the development of clinically viable furin inhibitors with improved specificity will be crucial before translation into patient care can be realized. Given the broad physiological roles of furin in processing multiple proproteins, systemic furin inhibition may result in off-target effects, highlighting the need for tumor-selective or pathway-focused therapeutic strategies that target downstream effectors such as TGF-β/ERK signaling rather than global furin blockade.

In summary, our study provides a new perspective on the oncogenic role of furin in CRC, revealing its coordinated regulation of both canonical and non-canonical pathways. Beyond its conventional role as a proprotein convertase, furin acts as a signaling enhancer, integrating multiple oncogenic cues that drive tumor growth, invasion, and chemoresistance. This expanded understanding of furin’s function highlights novel therapeutic opportunities, particularly through dual inhibition of the furin–TGF-β/ERK axis, which may help limit tumor progression and remodel the tumor microenvironment toward a less aggressive phenotype.

## 5. Conclusions

Our work highlights furin as a central regulator of CRC progression and chemoresistance through its interaction with the TGF-β/ERK pathway. By establishing a positive feedback loop with TGF-β, furin sustains oncogenic signaling and promotes tumor aggressiveness. These findings provide a strong rationale for the development of furin-targeted therapies, either alone or in combination with existing chemotherapeutics, as a strategy to overcome drug resistance and improve clinical outcomes in CRC patients.

## Figures and Tables

**Table 1:** Correlation of furin expression with clinico-pathological parameters in colorectal carcinoma.

	Total		Furin High		Furin Low		*p* Value
	n	%	n	%	n	%	
**Total Number of Cases**	1131		530	46.9	601	53.1	
**Age**							
≤50 years	378	33.4	158	29.8	220	36.6	0.0156
>50 years	753	66.6	372	70.2	381	63.4	
**Sex**							
Male	598	52.9	276	52.1	322	53.6	0.6136
Female	533	47.1	254	47.9	279	46.4	
**Tumor Site**							
Left colon	917	81.1	422	79.6	495	82.4	0.2404
Right colon	214	18.9	108	20.4	106	17.6	
**Histological Type**							
Adenocarcinoma	1008	89.1	469	88.5	539	89.7	0.5201
Mucinous Carcinoma	123	10.9	61	11.5	62	10.3	
**pT**							
T1	46	4.1	17	3.3	29	4.9	0.1017
T2	178	16.0	80	15.4	98	16.5	
T3	771	69.4	376	72.6	395	66.6	
T4	116	10.5	45	8.7	71	12.0	
**pN**							
N0	579	52.1	259	50.1	320	53.8	0.3530
N1	329	29.6	155	30.0	174	29.2	
N2	204	18.3	103	19.9	101	17.0	
**pM**							
M0	975	86.8	449	85.5	526	88.0	0.2285
M1	148	13.2	76	14.5	72	12.0	
**Tumor Stage**							
I	177	15.7	71	13.4	106	17.7	0.1537
II	376	33.3	174	32.8	202	33.7	
III	427	37.8	208	39.3	219	36.6	
IV	148	13.2	76	14.5	72	12.0	
**Differentiation**							
Well differentiated	105	9.5	50	9.6	55	9.3	0.8306
Moderate differentiated	892	80.5	420	80.9	472	80.2	
Poor differentiated	111	10.0	49	9.5	62	10.5	
**MSI-IHC**							
MSI-H	107	9.5	49	9.3	58	9.7	0.8162
MSS	1024	90.5	481	90.7	543	90.3	
**pERK1/2 expression**							
High	413	38.0	240	45.5	173	31.0	<0.0001
Low	673	62.0	287	54.5	386	69.0	
**TGFβ1 expression**							
High	323	30.7	248	55.1	75	12.5	<0.0001
Low	728	69.3	202	44.9	526	87.5	
***BRAF* mutation**							
Present	36	3.2	22	4.2	14	2.4	0.0873
Absent	1074	96.8	501	95.8	573	97.6	
***KRAS* mutation**							
Present	412	36.9	226	43.1	186	31.5	<0.0001
Absent	704	63.1	299	56.9	405	68.5	

## Data Availability

All data generated or analyzed during this study are included in this published article.

## Supplementary Materials

The following supporting information can be downloaded at: https://www.mdpi.com/article/10.3390/cells15010043/s1, Table S1: IC_50_ values for parental and 5-FuR CRC cell lines; Figure S1: Uncropped Western blot images; Figure S2: Immunohistochemistry analysis of furin in normal colonic mucosa; Figure S3: Effect of furin on Smad2/3 phosphorylation.
