# Supplementary material for: Furin Drives Colorectal Cancer Progression and Chemoresistance Through the TGF-β/ERK Signaling Pathway
Source: Cells. 2025 Dec 25;15(1):43. doi: 10.3390/cells15010043 (PMC12785517; doi:10.3390/cells15010043)
Supplement: Supplementary file 1 [file cells-15-00043-s001.zip › cells-4046224-supplementary.pdf]

Table S1. IC_{50} values for parental and 5-FuR CRC cell lines

Cell line	IC_{50} Parental (μM)	IC_{50} 5-FuR (μM)
HCT116	87.56 ± 10	160.05 ± 9.8
DLD1	88.29 ± 4.1	143.97 ± 3.2
LOVO	77.70 ± 5.4	150.94 ± 15.6

Figure S1. Uncropped Western blot Images

Figure 2C

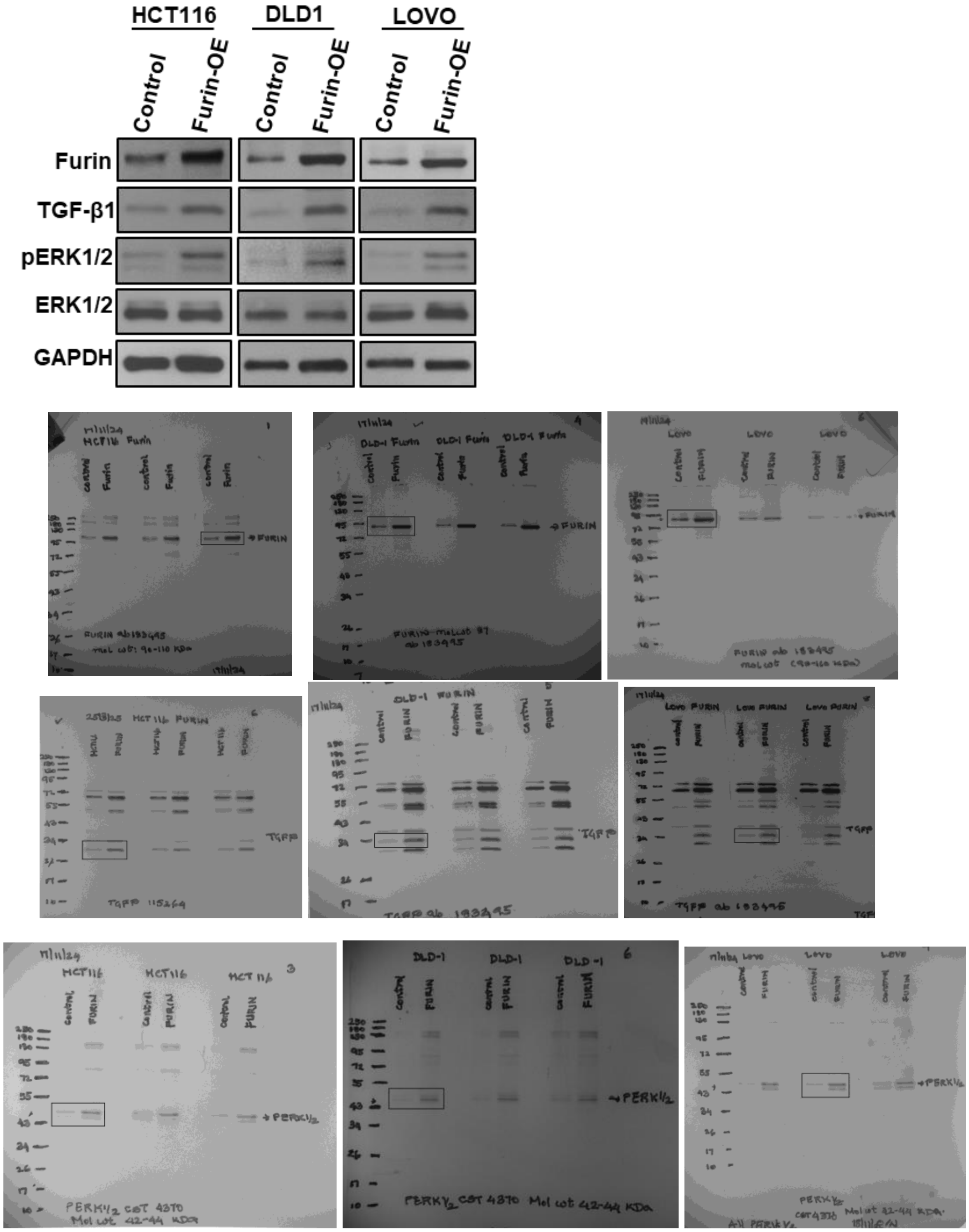

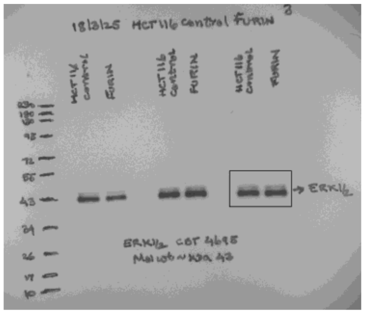
18/3/25 HCT116 control FURIN
HCT116 control
FURIN
ERK1/2
ERK1/2 CST 4695
Mol wt ~kDa 43

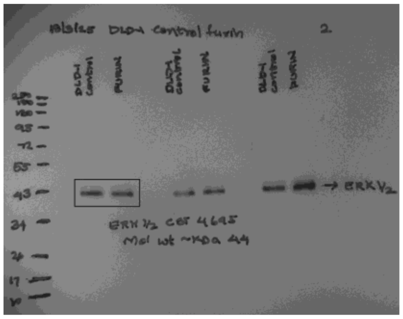
18/3/25 DLD-1 control furin
DLD-1 control
FURIN
ERK1/2
ERK 1/2 CST 4695
Mol wt ~kDa 44

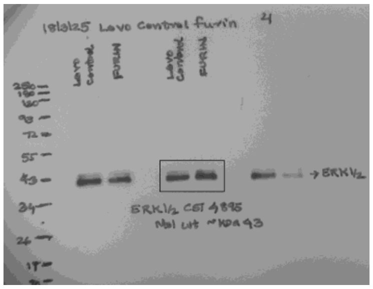
18/3/25 Lovo control furin
Lovo control
FURIN
ERK1/2
ERK1/2 CST 4695
Mol wt ~kDa 43

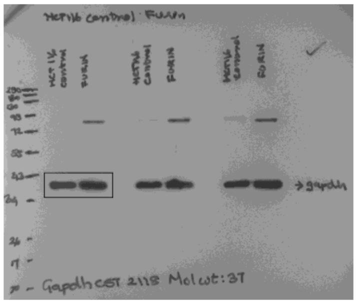
HCT116 control FURIN
HCT116 control
FURIN
gapdh
Gapdh CST 2118 Mol wt: 37

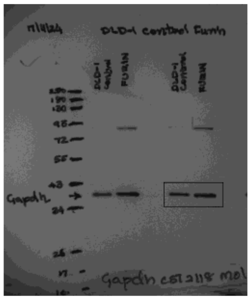
DLD-1 control FURIN
DLD-1 control
FURIN
Gapdh
Gapdh CST 2118

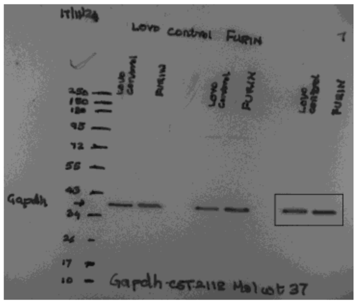
Lovo control FURIN
Lovo control
FURIN
Gapdh
Gapdh CST 2118 Mol wt 37

Figure 2E

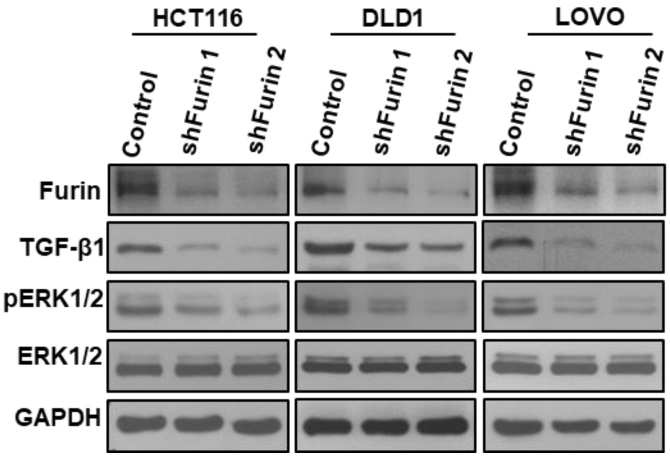
HCT116
DLD1
LOVO
Control
shFurin 1
shFurin 2
Control
shFurin 1
shFurin 2
Control
shFurin 1
shFurin 2
Furin
TGF-β1
pERK1/2
ERK1/2
GAPDH

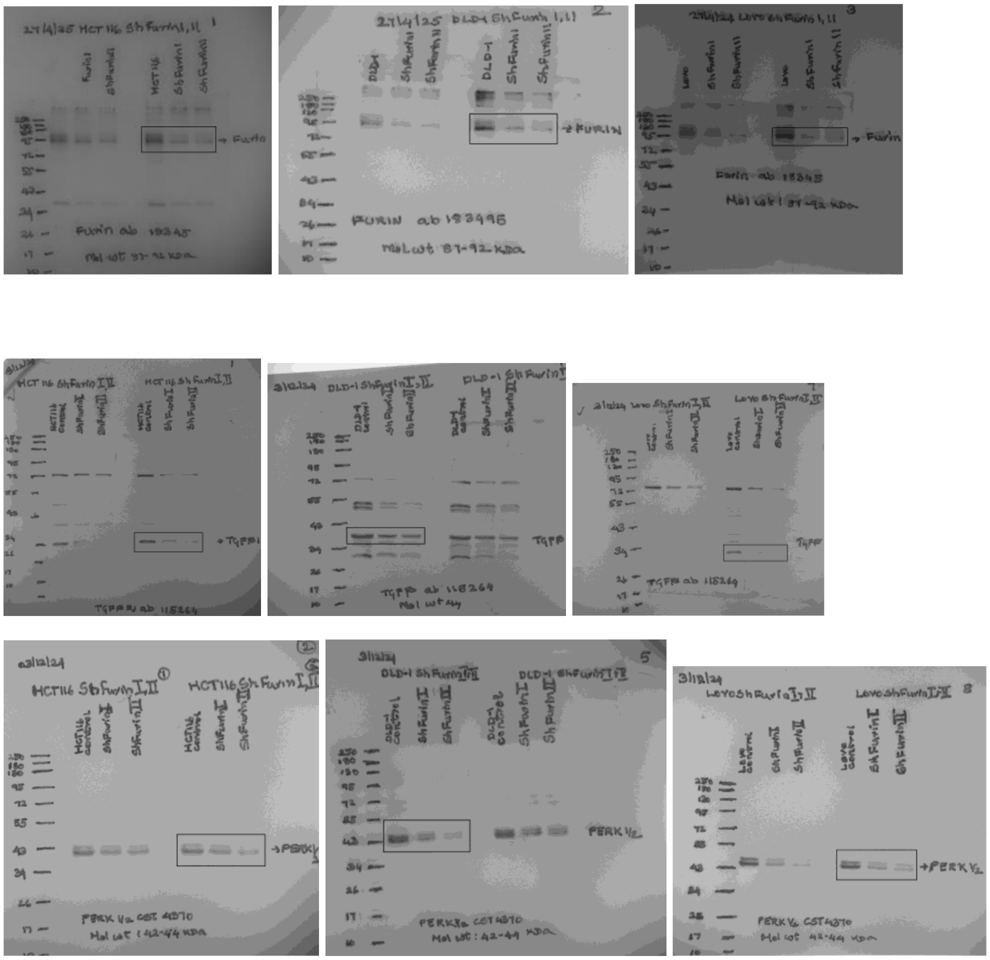

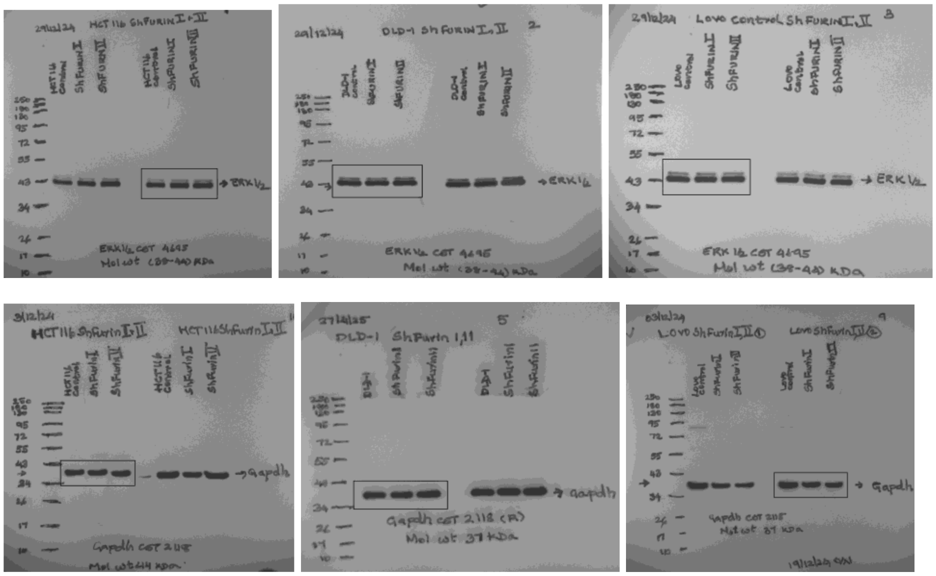

ERK1/2
Gapdh
Mol wt 37 KDa

Figure 3A

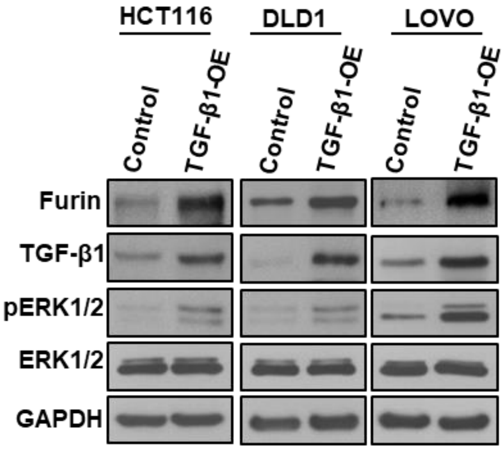
HCT116
DLD1
LOVO
Control
TGF-β1-OE
Control
TGF-β1-OE
Control
TGF-β1-OE
Furin
TGF-β1
pERK1/2
ERK1/2
GAPDH

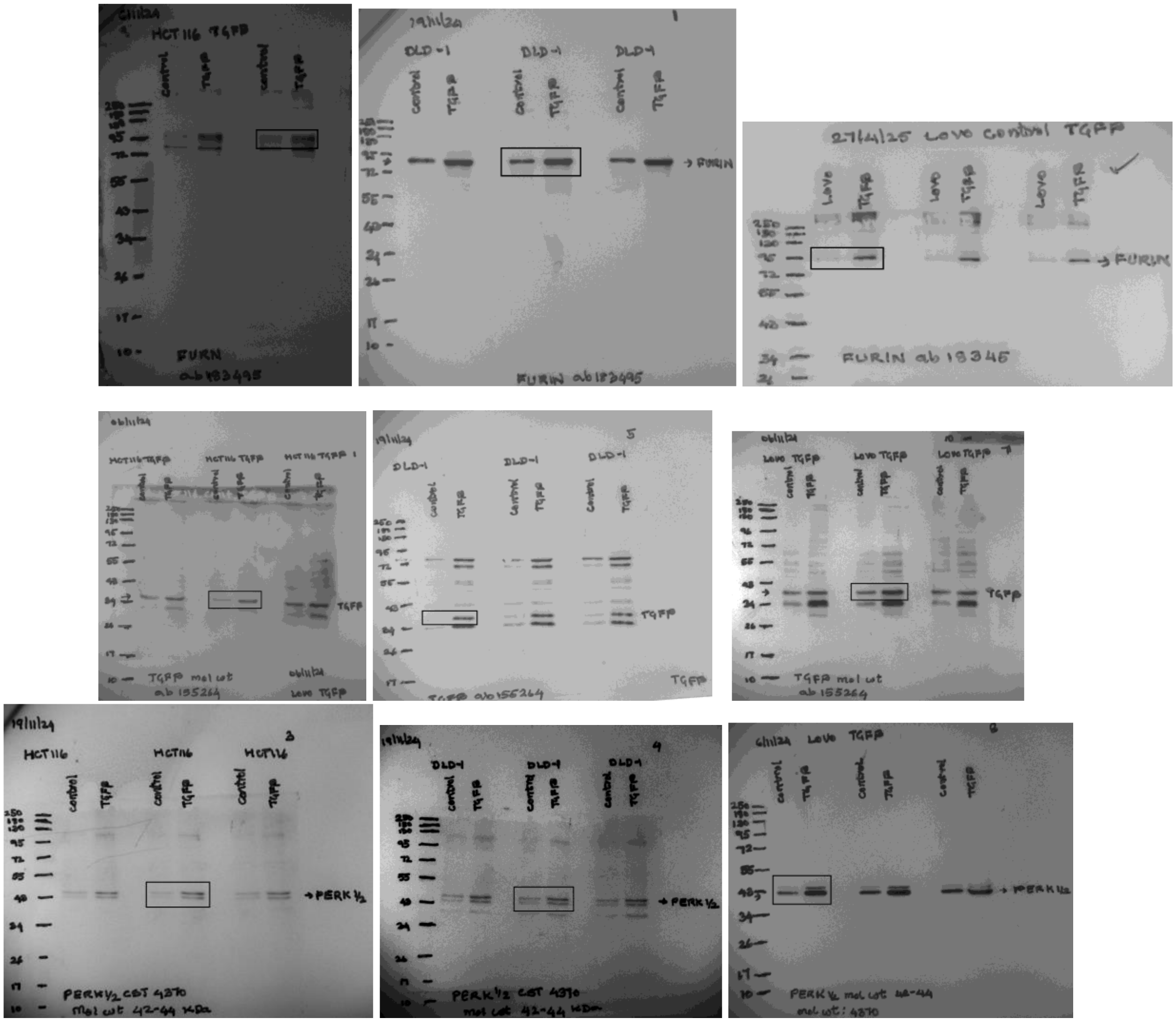

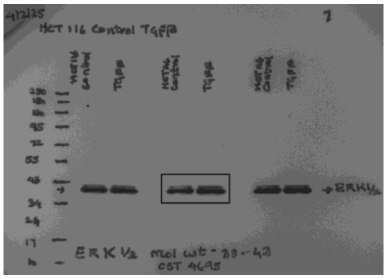
4/2/25
HCT 116 Control TGFβ
ERK1/2
ERK1/2 mol wt: 39-42
CST 4695

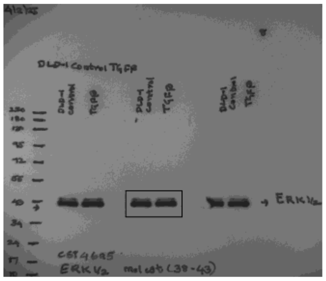
4/2/25
DLD-1 Control TGFβ
ERK1/2
CST 4695
ERK1/2 mol wt (39-43)

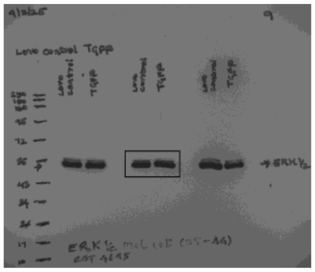
4/2/25
Lovo control TGFβ
ERK1/2
ERK1/2 mol wt (35-44)
CST 4695

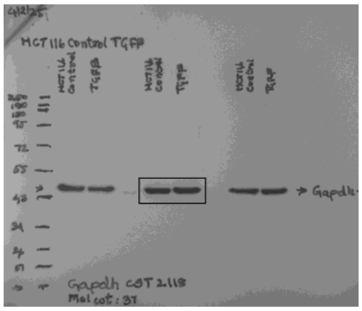
4/2/25
HCT 116 Control TGFβ
Gapdh
Gapdh CST 2118
Mol wt: 37

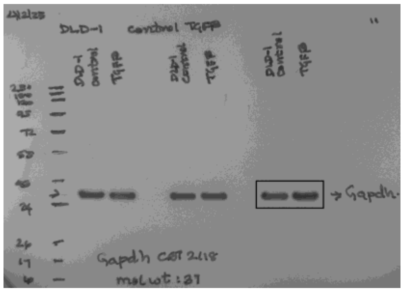
4/2/25
DLD-1 Control TGFβ
Gapdh
Gapdh CST 2118
mol wt: 37

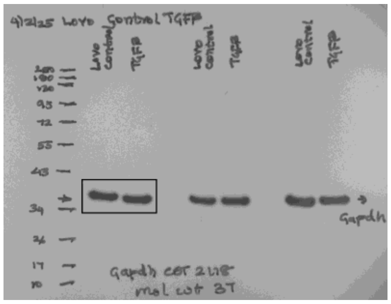
4/2/25 Lovo Control TGFβ
Gapdh
Gapdh CST 2118
mol wt 37

Figure 3B

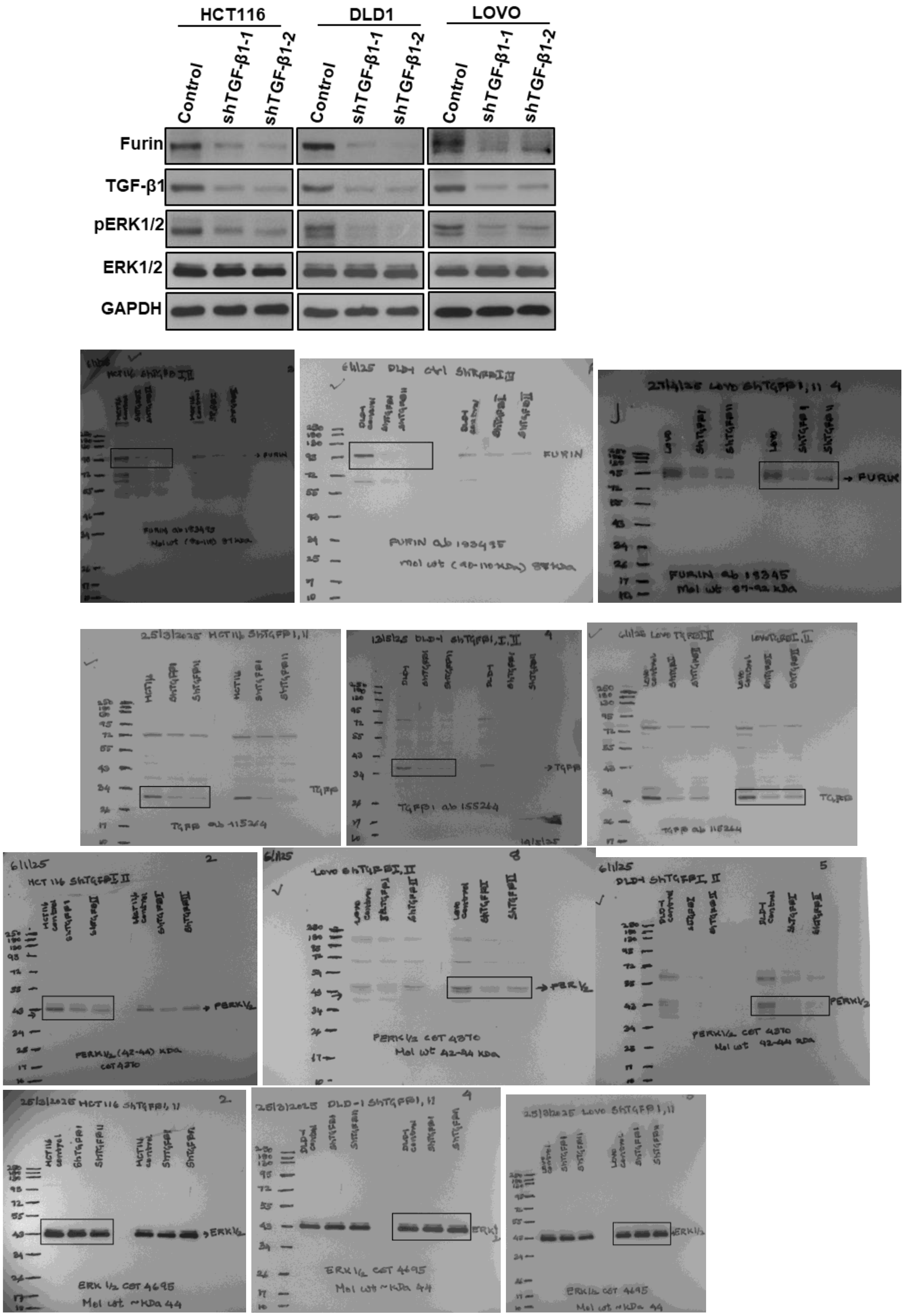
HCT116
DLD1
LOVO
Control
shTGF-β1-1
shTGF-β1-2
Furin
TGF-β1
pERK1/2
ERK1/2
GAPDH

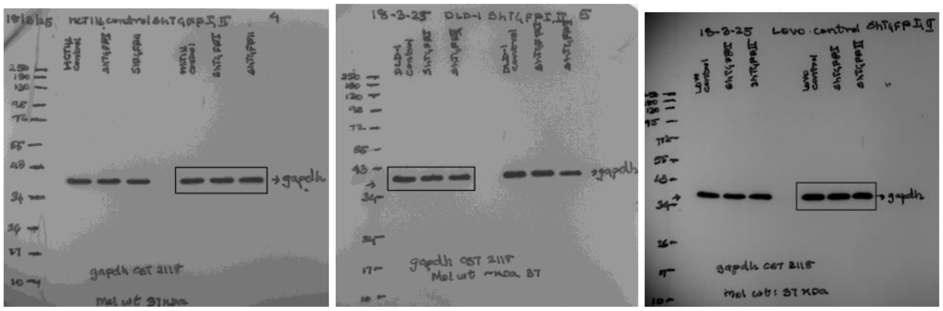
gapdh
gapdh
gapdh

Figure 4D

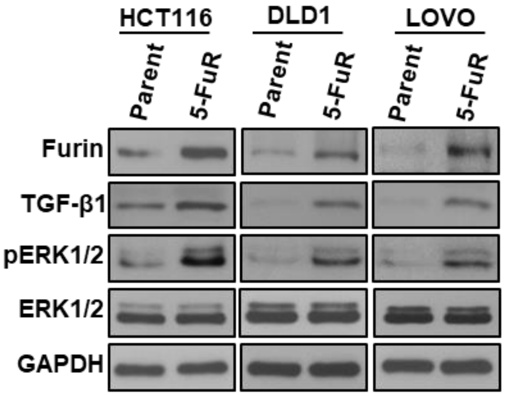
HCT116
DLD1
LOVO
Parent
5-FuR
Parent
5-FuR
Parent
5-FuR
Furin
TGF-β1
pERK1/2
ERK1/2
GAPDH

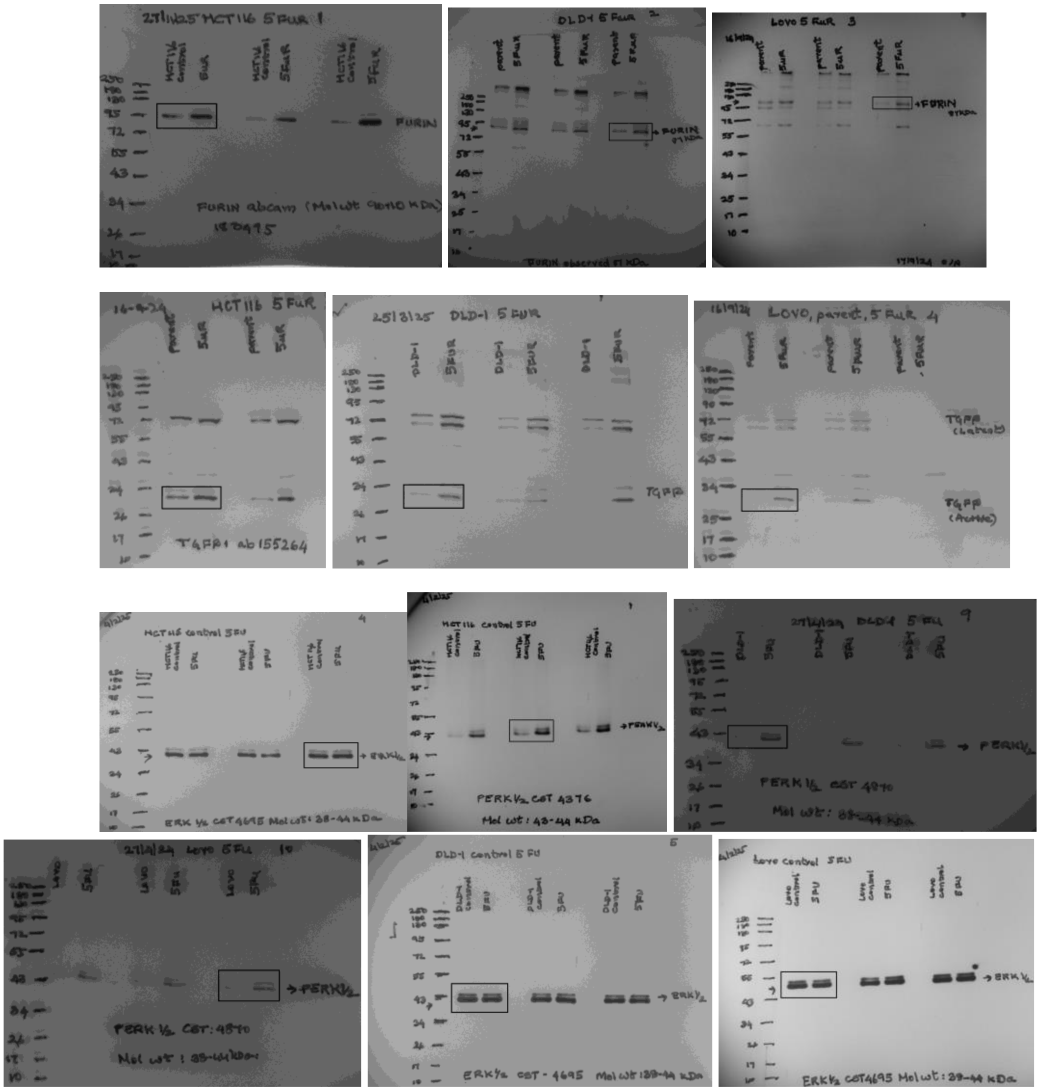

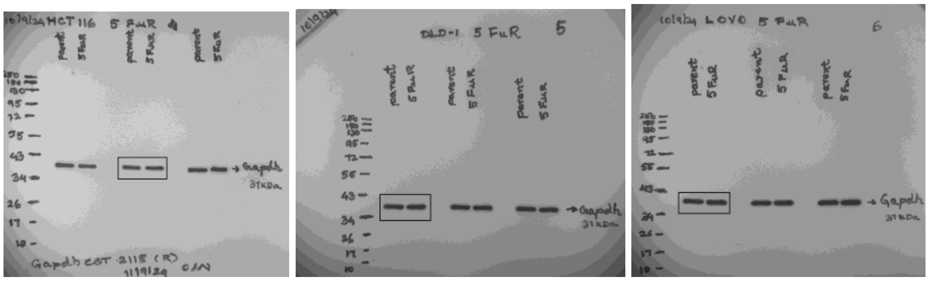

Figure 5D

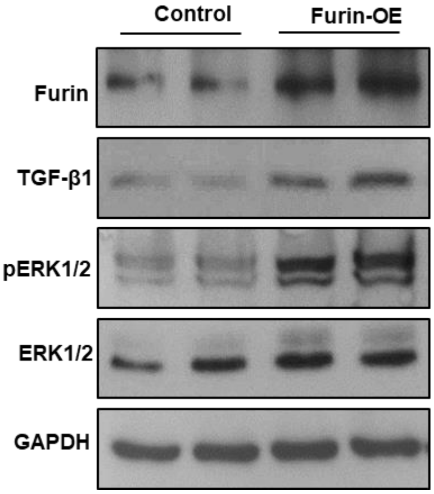

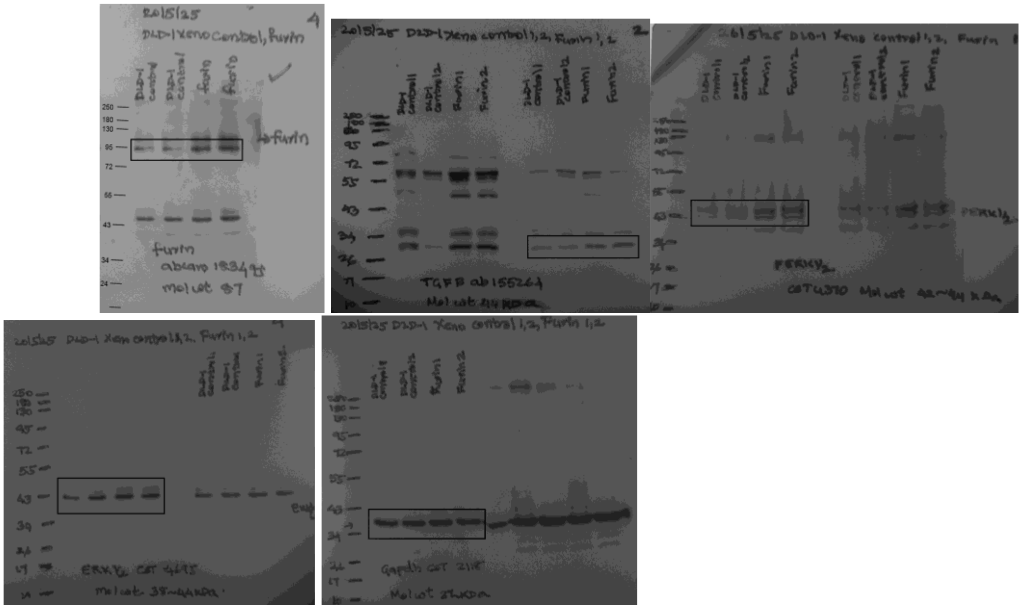

Figure 6D

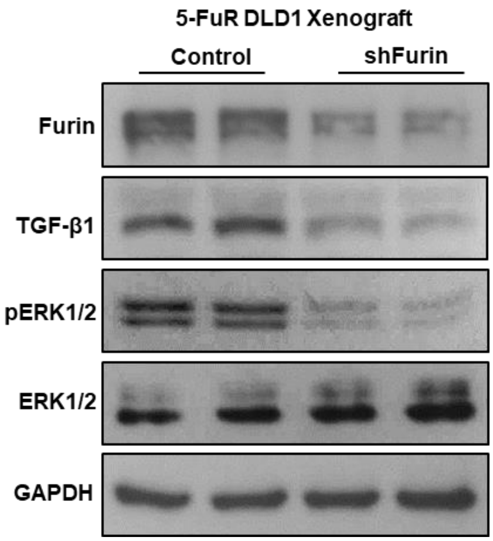
5-FuR DLD1 Xenograft
Control
shFurin
Furin
TGF-β1
pERK1/2
ERK1/2
GAPDH

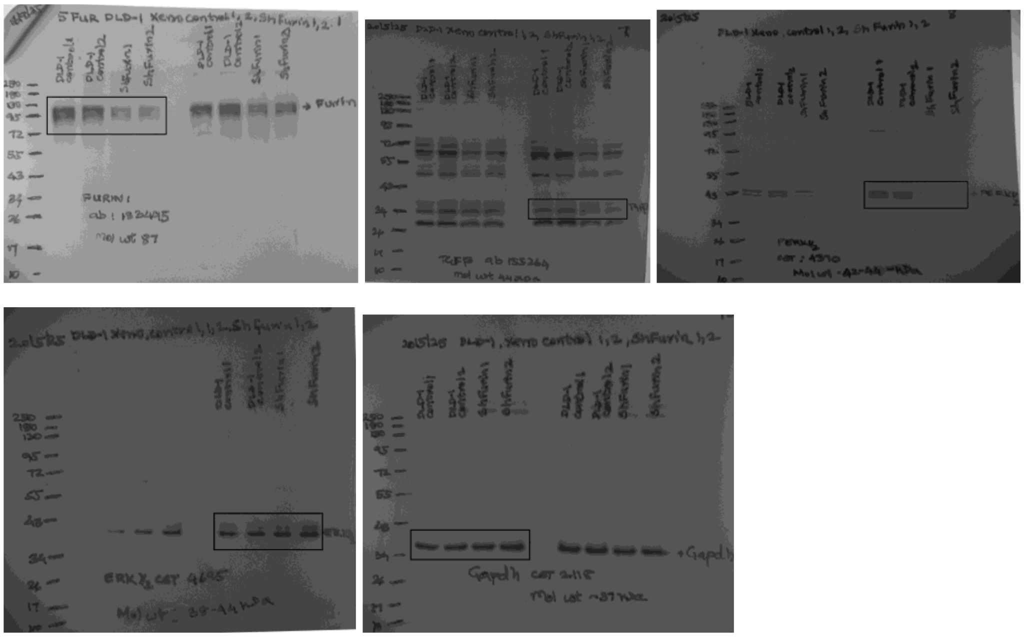

Supplementary Figure S2. Immunohistochemistry analysis of furin in normal colonic mucosa

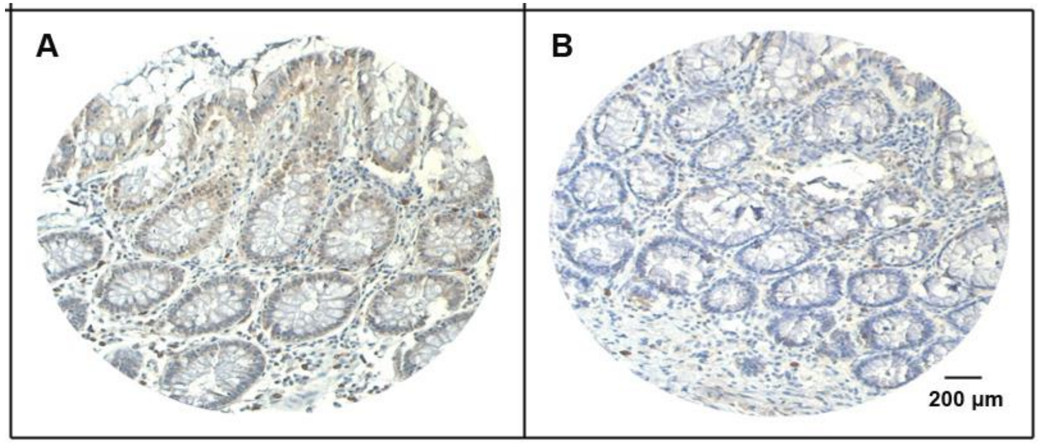

Figure S2. Tissue microarray (TMA) based immunohistochemistry analysis of furin in normal colonic mucosa. (A) shows adjacent normal colonic mucosa stained with anti-Furin antibody. (B) the negative control slide processed without primary antibody. 20 X/0.70 objective on an Olympus BX 51 microscope (Olympus America Inc, Center Valley, PA, USA, scale bar=200 μm).

Figure S3. Effect of furin on Smad2/3 phosphorylation

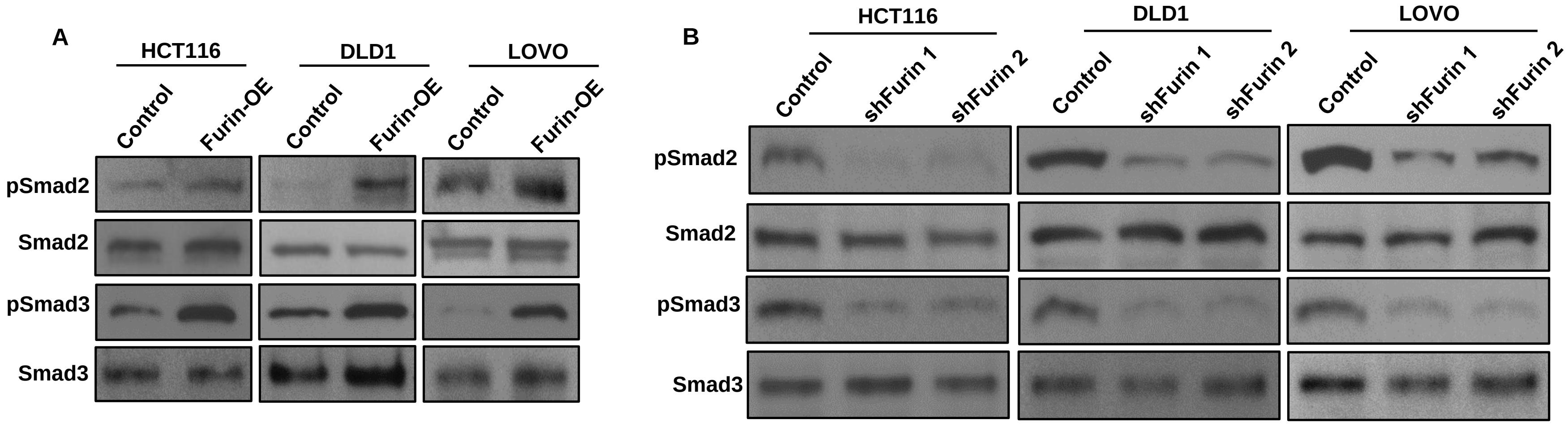

(A) Furin overexpression activates Smad2/3 phosphorylation. Protein lysates from furin-overexpressing clones were analyzed by immunoblotting. **(B)** Silencing furin decreases Smad2/3 phosphorylation. Protein lysates from furin-knockdown clones were analyzed by immunoblotting.
